# Appropriate antibiotic management of bacterial lower respiratory tract infections

**DOI:** 10.12688/f1000research.14226.1

**Published:** 2018-07-23

**Authors:** Charles Feldman, Guy Richards

**Affiliations:** 1 Department of Internal Medicine, Faculty of Health Sciences, University of the Witwatersrand, 7 York Road, Parktown, Johannesburg, 2193, South Africa; 2 Division of Critical Care, Charlotte Maxeke Johannesburg Academic Hospital, and Faculty of Health Sciences, University of Witwatersrand, 7 York Road, Parktown, Johannesburg, 2193, South Africa

**Keywords:** antibiotics, chronic obstructive pulmonary disease, community-acquired pneumonia, antimicrobial stewardship

## Abstract

Lower respiratory tract infections are the leading cause of infectious disease deaths worldwide and are the fifth leading cause of death overall. This is despite conditions such as pneumococcal infections and influenza being largely preventable with the use of appropriate vaccines. The mainstay of treatment for the most important bacterial lower respiratory tract infections, namely acute exacerbations of chronic obstructive pulmonary disease (AECOPD) and community-acquired pneumonia (CAP), is the use of antibiotics. Yet despite a number of recent publications, including clinical studies as well as several systematic literature reviews and meta-analyses, there is considerable ongoing controversy as to what the most appropriate antibiotics are for the empiric therapy of CAP in the different settings (outpatient, inpatient, and intensive care unit). Furthermore, in the case of AECOPD, there is a need for consideration of which of these exacerbations actually need antibiotic treatment. This article describes these issues and makes suggestions for appropriately managing these conditions, in the setting of the need for antimicrobial stewardship initiatives designed to slow current emerging rates of antibiotic resistance, while improving patient outcomes.

## Introduction

Lower respiratory tract infections (LRTIs), which generally are considered to include acute bronchitis, bronchiolitis, influenza, and pneumonia, are a significant cause of morbidity and mortality in patients worldwide ^1, 2^. The Global Burden of Disease Study evaluated evidence for the global, regional, and national morbidity and mortality of LRTIs and indicated that, for 2015, LRTIs were the leading infectious disease cause of death and the fifth leading cause of death overall ^3^. They estimated that LRTIs caused 2.74 million deaths and 103.0 million disability-adjusted life years (DALYs). While the burden had decreased in children younger than 5 years of age, it had increased in many regions for individuals older than 70 years. The study specifically investigated four etiologies—two bacterial and two viral—and noted that pneumococcal pneumonia was the most common etiology, which led to 1,517,388 deaths or 55.4% of LRTI deaths in all ages. Pneumococcal pneumonia was also a cause of a significant number of deaths in the elderly population worldwide (693,041 deaths in adults at least 70 years old). LRTIs are largely preventable causes of death, as vaccines are available against both influenza and pneumococcal pneumonia. Furthermore, while antiviral therapy is available for the treatment of influenza infections once they occur, the mainstay of treatment for community-acquired pneumonia (CAP) and acute bacterial exacerbations of chronic obstructive pulmonary disease (COPD), which are the focus of this overview of the recent literature, is the use of antibiotics.

Overuse of antibiotics both in and out of hospital has resulted in an exponential increase in resistance globally. This has, and will, impact upon the ability to treat infections and be directly associated with increasing morbidity and mortality ^4, 5^. This has resulted in numerous antimicrobial stewardship (AMS) programs—both in South Africa and internationally—designed to slow the rate at which organisms develop resistance and at the same time improve outcomes ^6, 7^. AMS programs consist of two major pillars: first, infection prevention and control and then appropriate use of antibiotics, the latter of which will be the focus of this article. Appropriate use implies the correct indication, dose, duration, and administration method (that is, according to pharmacokinetic principles).

The actual antibiotic choice for bacterial LRTIs depends upon the likely organism; however, it is recognized that distinguishing viral from bacterial infections, both in CAP and in acute exacerbations of COPD, and recognizing non-infective COPD exacerbations are not always clear-cut processes. In fact, as many as 60% of COPD exacerbations may be due to viral infections, in particular rhinovirus, and in winter the influenza virus, and there is an increasing recognition of the important role for viruses in the etiology of CAP ^8, 9^.

Although they are beyond the scope of this article, a brief review of biomarkers and the role they may play in the decision regarding the need for initiation, discontinuation, or duration of antibiotic treatment are of interest. Of the various biomarkers described, perhaps the most studied are C-reactive protein (CRP) and procalcitonin (PCT). One systematic review and meta-analysis evaluated the use of CRP to guide antibiotic therapy in patients presenting to primary care with symptoms of acute respiratory infections and reported a significant reduction in antibiotic use with a slight increase in hospital admissions ^10^. The European guideline on LRTIs indicates that out of the hospital setting, where chest radiographic confirmation of CAP is usually not available, a measurement of CRP in a patient suspected of having CAP can be performed (a point-of-care test is currently available) ^11^. They recommend that a level of less than 20 mg/L at presentation, in the presence of symptoms for at least 24 hours, makes pneumonia highly unlikely but that a level of more than 100 mg/L makes pneumonia likely.

With regard to PCT, there are some differences in the conclusions reached in the various studies. One systematic review and meta-analysis assessed the safety and efficacy of PCT for starting and stopping antibiotics in a range of patients with varying severity of acute respiratory tract infections in different clinical settings ^12^. The authors concluded that the use of PCT to guide initiation and duration of antibiotic treatment was associated with a lower risk of mortality, lower antibiotic consumption, and lower risk of antibiotic side effects. The authors, when using a patient-level meta-analysis, reached similar conclusions ^13^. However, a recent study of PCT-guided use of antibiotics in the treatment of patients with suspected LRTIs did not result in less use of antibiotics than did usual care ^14^. Furthermore, there is no point-of-care test available for the measurement of PCT, which is also costly.

## Antibiotic treatment of community-acquired pneumonia

Antibiotics are the mainstay of therapy for CAP, and the initial antibiotic treatment needs to be empiric, as the causative organism or organisms are unknown at the time of presentation. However, there has been ongoing debate over a considerable period of time as to the most appropriate choice of initial empiric antibiotic treatment in the different settings: outpatient, inpatient, and intensive care unit (ICU). A number of national and international guidelines, which describe the appropriate management of CAP, have been developed; some of these have been updated recently or are in the process of being updated ^11, 15, 16^. It is clear when evaluating the guidelines that differences exist with regard to the various recommendations, including those for initial empiric antibiotic therapy ^11, 15– 17^.

For outpatient antibiotic therapy of CAP, the Infectious Diseases Society of America/American Thoracic Society (IDSA/ATS) guideline recommends a macrolide or tetracycline for previously healthy patients with no risk factors for drug-resistant *Streptococcus pneumoniae* (DRSP) infections. Furthermore, a respiratory fluoroquinolone or a beta-lactam plus a macrolide is recommended in the presence of certain comorbidities or risk factors for DRSP infections ^15^. This is in contrast with the European guideline, which recommends amoxicillin or tetracycline for outpatient use with a macrolide or tetracycline being used only in the case of penicillin allergy, in settings with low levels of pneumococcal macrolide resistance ^11^. Similarly, the South African guideline recommends amoxicillin as the treatment of choice, and a macrolide is to be used in the case of penicillin allergy in settings with low levels of pneumococcal macrolide resistance and other options are reserved for the elderly or for those with comorbidities or recent antibiotic use or both ^16^. A recent literature review from Europe described the etiology and management of CAP in adults, which included both primary care and hospitalized cases ^18^. The authors noted differences in antibiotic prescribing habits in the various regions of Europe. Beta-lactams were the most commonly prescribed class of antibiotics, and monotherapy was more common than combination therapy, but hospitalized patients more commonly received combination therapy than did outpatients. These differences in antibiotic recommendations could be ascribed to differences in the microbial etiology of CAP in the different regions, differences in the prevalence of antibiotic-resistant pathogens, differing patient populations, differences in national guideline recommendations, and other local factors, including regulatory requirements. A recent Cochrane review concluded that there was insufficient evidence from randomized controlled trials to make evidence-based recommendations regarding appropriate treatment in adult outpatients with CAP ^19^. Owing to the low number of studies comparing the same antibiotic pairs, pooling of the data was also not possible; in general, the individual studies did not suggest any significant differences in the efficacy of the various antibiotics studied.

For inpatients with CAP, the IDSA/ATS guideline recommends the use of either a beta-lactam-macrolide combination or fluoroquinolone monotherapy for non-critically ill cases ^15^; the European and South African guidelines have the addition of a macrolide to the beta-lactam as an option ^11, 16^. Most of the guidelines recommend a combination therapy of a beta-lactam and macrolide or fluoroquinolone in those requiring ICU admission, and additional options are for possible pseudomonal infection ^11, 15, 16, 20^. The most controversial area has been whether the use of a macrolide antibiotic should be an obligatory component of initial antibiotic treatment ^21^. Earlier studies of hospitalized patients with moderately severe pneumonia suggested that beta-lactam monotherapy was not inferior to beta-lactam–macrolide combination therapy ^22, 23^. However, as has been indicated by several investigators, those studies had certain limitations that make it difficult to accept their conclusions ^20, 21^. A recent review highlighted all the studies documenting a benefit of combination therapy among inpatients; most of these studies were among CAP patients hospitalized in the ward and not the ICU ^17^. Furthermore, Okumura *et al.* documented that in hospitalized CAP patients at low risk of drug-resistant pathogens, beta-lactam–macrolide combination treatment lowered 30-day mortality compared with beta-lactam therapy alone: adjusted odds ratio (OR) 0.28, 95% confidence interval (CI) 0.09–0.87 ^24^.

There may still be some debate as to the need for adding a macrolide in less severely ill hospitalized cases. However, two matched case-control studies were published by the CAPUCI II Consortium on severe CAP patients admitted to the ICU ^25, 26^. In the first, Gattarello *et al.* compared the outcome of two cohorts of critically ill patients with pneumococcal CAP from different time periods (2001–2002 and 2008–2013) ^25^. The investigators noted that the mortality rate decreased by 18% from the earlier to the later cohort (together with other outcome benefits). This was determined on multivariate analysis to be associated with giving of the first dose of antibiotic within 3 hours (OR 0.36, 95% CI 0.15–0.87) and to the use of combination therapy (OR 0.19, 95% CI 0.07–0.51), which occurred more commonly in the second time period. The most frequently used antibiotic regimen was the combination of a cephalosporin with a macrolide, but other combinations included a cephalosporin and a fluoroquinolone. The second study was on patients with severe non-pneumococcal CAP, and the results were essentially similar ^26^. Once again, the most commonly used antibiotic regimen was a cephalosporin and a macrolide. Early antibiotic treatment (OR 0.07, 95% CI 0.02–0.22) and combined antibiotic therapy (OR 0.23, 95% CI 0.07–0.74), which occurred more commonly in the second time period, were independently associated with a lower ICU mortality.

Whereas a few studies have documented that combination therapy with a beta-lactam and a macrolide or fluoroquinolone has no additional benefit in critically ill patients with CAP ^27, 28^, several recent studies have confirmed the benefit of combination therapy in this situation ^29– 31^. For example, Pereira *et al.* documented that combination antibiotic therapy together with a macrolide was independently associated with a reduction in hospital stay (OR 0.17, 95% CI 0.06–0.51) and 6-month mortality (OR 0.21, 95% CI 0.07–0.57) ^31^.

With regard to systematic reviews and meta-analyses of antibiotic therapy in critically ill patients with CAP, an early study concluded that there was a significant reduction in mortality when macrolides were used as part of treatment: 21% (836/4,036) versus 24% (1,369/5,814), risk ratio 0.82, 95% CI 0.70–0.97, *p* = 0.02 ^32^. When macrolide monotherapy was excluded, the mortality benefit of macrolides was still maintained. There was a trend towards better mortality with beta-lactam–macrolide therapy compared with beta-lactam–fluoroquinolone therapy: mortality with beta-lactam–macrolide therapy 20% (511/2,561 patients) versus 23% (386/1,680) with beta-lactam–fluoroquinolone therapy (risk ratio 0.83, 95% CI 0.67–1.03, *p* = 0.09) ^32^. A number of more recent systematic reviews of antibiotic therapy in patients with CAP have been undertaken. Some that did not restrict the study to severely ill cases documented no benefit on 30-day mortality of beta-lactam–macrolide or beta-lactam–fluoroquinolone combination therapies over fluoroquinolone monotherapy ^33, 34^. However, Horita *et al.* concluded that, compared with beta-lactam monotherapy, combination therapy with a beta-lactam plus macrolide may decrease all-cause mortality only in severe CAP ^35^. The authors did recommend caution in this interpretation, as the conclusion was based mainly on observational studies. Lastly, Lee *et al.*, in their systematic review and meta-analysis of patients with severe CAP, noted that the overall mortality of the beta-lactam–macrolide group was lower than that of the beta-lactam–fluoroquinolone group (19.4% versus 26.8%) (OR 0.68, 95% CI 0.49–0.94) ^36^. Furthermore, length of hospital stay was shorter in the former group compared with the latter group, although there was no difference in length of ICU stay. However, despite these positive findings, the authors did indicate the need for caution with the conclusions because of the high risk of bias in the trials and methodological limitations.

The reason for the potential benefit of macrolide combination therapy in patients with CAP is unclear, but it is known that macrolide antibiotics have additional anti-inflammatory, immunomodulatory effects and do not directly lyse bacteria, which may play an important role. In the case of the pneumococcal infections, for example, lytic antibiotics increase the release of the pro-inflammatory toxin pneumolysin as well as cell-wall components, which may be associated with host tissue injury ^37^. For this reason, other investigators have studied the timing of combination antibiotic treatment ^38, 39^. Such investigators have theorized that administering macrolides some time prior to the beta-lactam agent may improve patient outcomes because of the anti-inflammatory effects that attenuate the inflammatory response initiated by the beta-lactam-induced lysis of bacteria. Metersky *et al.* undertook a retrospective cohort study using electronic health records from a large database, comparing the outcome of CAP cases receiving a macrolide 1 hour before a cephalosporin compared with cases receiving a cephalosporin 1 hour before the macrolide ^38^. The adjusted mortality was about 30% lower in the former group compared with the latter group, although this did not reach statistical significance. There were also trends towards lower combined in-hospital mortality/hospice discharge and reduced length of stay. The authors concluded that it was worth pursuing this investigation with a larger cohort and perhaps in subsets of severe pneumonia cases. Furthermore, Peyrani *et al.* undertook a secondary analysis of data from the Community-Acquired Pneumonia Organisation (CAPO) database ^39^. They documented that, in CAP cases in whom a macrolide had been administered before the beta-lactam, compared with the reverse, the time to clinical stability (3 versus 4 days, *p* = 0.011), length of hospital stay (6 versus 7 days, *p* = 0.002) and mortality (3% versus 7.2%, *p* = 0.228) were lower.

Based on the findings of the majority of the studies, and in line with the recommendations of other investigators ^21^, we would recommend that a combination of a beta-lactam and a macrolide be used in hospitalized and severely ill cases with CAP and that the macrolide be given prior to initiation of the beta-lactam. Furthermore, the antibiotics should be started as soon as possible after confirmation of the diagnosis of CAP. With regard to the fluoroquinolones, careful consideration needs to be given to their routine use for suspected CAP in areas in which tuberculosis (TB) is endemic because it is frequently difficult to clinically differentiate TB from CAP on initial presentation of patients ^40^. Therefore, there is concern that the use of fluoroquinolone in someone with TB but suspected of having CAP may lead to a delay in the diagnosis of TB and, moreover, be associated with the development of drug-resistant TB ^40, 41^. Clearly, the above discussion has focused on empiric antibiotic therapy, and once the results of microbiological testing become available, antibiotic treatment should be tailored appropriately to the findings.

Two additional aspects of antibiotic therapy in patients with CAP need mention. The first is the importance of time to initiation/administration of antibiotics relative to the time of presentation with CAP. Although there has been the odd study suggesting that time to initiation of antibiotics has no impact on various patient outcomes ^42^, this contention is not supported by the majority of additional studies ^25, 26, 43, 44^. All of the studies from the CAPUCI II Consortium of critically ill patients with CAP indicated that early antibiotic administration (<3 hours) was independently associated with a lower ICU mortality ^25, 26, 43^. An additional study of hospitalized patients with CAP indicated that delay of the first dose of antibiotics beyond 4 hours was one of the independent predictors of mortality (adjusted OR 3.9) ^44^. Furthermore, the systematic review by Lee *et al.* ^34^, assessing three aspects of antibiotic therapy in hospitalized patients with CAP, noted that administration of antibiotics within 4 to 8 hours of hospital arrival was associated with a reduction in mortality, although the quality of evidence was assessed as being relatively poor. Thus, while it is clear that patients with severe CAP (especially those with septic shock) should receive antibiotics as soon as possible ^20^, the impact of time to antibiotic initiation on outcome in less severely ill cases is not clear ^20^. It seems prudent to initiate antibiotics as soon as possible in patients suspected of having CAP; however, it should not be at the expense of an adequate consideration of possible alternative diagnoses, including acute bronchitis, influenza, pulmonary embolism, and heart failure ^20^. In such patients, administration of an antibiotic would have no benefit, would be associated with potentially serious consequences, and would be contrary to AMS initiatives.

Lastly, some consideration should be given to the appropriate duration of antibiotic therapy for patients with CAP which has varied over time, even though earlier studies and even two meta-analyses indicated that shorter duration of therapy (for example, 7 days or less) could be safely and effectively used in patients with mild to moderately severe pneumonia ^45^. One recent multicenter non-inferiority randomized controlled trial of hospitalized patients with CAP randomly assigned patients at day 5 to an intervention or to a control group. The former were treated with antibiotics for a minimum of 5 days, and the antibiotic was stopped when the temperature had been 37.8°C or less for 48 hours and the patients had no more than one CAP-associated sign of clinical instability (this being in line with the recommendation of the IDSA/ATS guideline on CAP management) ^46^. The antibiotic treatment in the control group of patients was determined by the individual patient’s physician. The results demonstrated non-inferiority of the shorter antibiotic course and supported the IDSA/ATS recommendations. In terms of potential limitations of the study, 60% of the patients were in low Pneumonia Severity Index (PSI) risk groups I–III and had a predicted mortality of less than 1%, and also there was a low rate of comorbid illnesses in the study population, which would limit generalizability to patients with significant comorbidity.

However, conversely, a more recent multicenter, non-inferiority, randomized controlled trial was undertaken in hospitalized CAP patients who had reached clinical stability within 5 days of hospitalization, who were randomly assigned to a standard or individualized group ^47^. The latter had antibiotics discontinued 48 hours after reaching clinical stability, having had at least 5 days of antibiotics. The study was stopped early because of apparent inferiority of the individualized treatment over the standard treatment with regard to the primary outcome, which was early failure within 30 days. This difference (11.2% in the individualized group versus 7.4% in the standard group) was not statistically significant, but the safety committee interrupted the study because at the time this was the first study to evaluate clinical stability as a proxy to shorten antibiotic exposure in patients hospitalized with CAP and the 30-day mortality was higher in the individualized versus the standard group.

However, other studies and reviews have supported shorter duration of antibiotic therapy in patients with CAP and suggested that the use of biomarkers, such as PCT, may be useful in guiding both the initiation and the duration of antibiotic treatment ^48, 49^. One group of investigators documented that an implementation strategy, tailored to identify previous barriers to early switch, was associated with a reduced duration of intravenous therapy ^50^. Another implemented a dedicated CAP team to manage low-risk, hospitalized CAP patients, which resulted in a reduced hospital length of stay, time to switch from intravenous antibiotics, and antibiotic duration without any adverse events ^51^.

It is interesting to briefly mention non-antibiotic adjunctive therapies, which—though clearly beyond the scope of this review—continue to be evaluated in critically ill patients with CAP, in whom the mortality remains high despite apparently appropriate antibiotic treatment. A number of such therapies have been studied, of which the use of corticosteroids (CSs) appears to be most promising, and a number of positive randomized controlled trials and systematic literature reviews have been published in recent years ^52, 53^. Most recently, the Cochrane database of systematic reviews updated its 2011 review of randomized controlled trials of systemic CS therapy, as adjunct to antibiotic therapy versus placebo or no CS, and concluded that CS therapy significantly reduces morbidity (various end-points) and mortality (relative risk 0.58, 95% CI 0.40–0.84) in adults with severe CAP; the number needed to treat for additional beneficial outcome was 18 patients (95% CI 12–49) to prevent one death ^54^. CSs also significantly reduced morbidity, but not mortality, for non-severe CAP in adults, and although there were more adverse events in the CS group (especially hyperglycemia), the harms did not outweigh the benefits. The South African CAP guideline makes some specific recommendations for the use of CSs in patients with severe CAP ^16^, and it would seem likely that as the older CAP guidelines are updated, more specific recommendations regarding adjunctive therapy for CAP will be included.

## Antibiotics for acute exacerbations of chronic obstructive pulmonary disease

**Figure 1.**
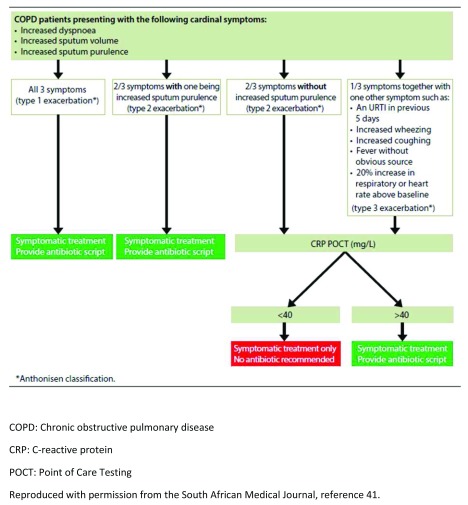
Recommendations for antibiotic use in non-hospitalized patients with acute exacerbations of chronic obstructive pulmonary disease. COPD, chronic obstructive pulmonary disease; CRP, C-reactive protein; POCT, point-of-care testing. Reproduced with permission from the *South African Medical Journal* ^41^.

Antibiotics are used in two instances in COPD: in order to treat an infection associated with an acute exacerbation (acute exacerbations of COPD [AECOPD]) and for prophylaxis. We have previously described a treatment algorithm designed to limit antibiotic use and assist clinicians in the outpatient setting. This algorithm was based upon severity of symptoms according to the Anthonisen criteria and point-of-care testing using the CRP where the clinical criteria are equivocal regarding severity ^55, 56^. Although the study by Anthonisen *et al.* ^56^ is old, various other studies have confirmed the value of antibiotics, particularly in the severe exacerbation; however, perhaps the most important of these criteria is sputum purulence and, as mentioned above, the presence of an elevated CRP ^55, 57– 60^ ( Figure 1). In addition, more recently, a meta-analysis that included four trials and 679 patients found that the use of PCT significantly reduced antibiotic use with an OR of 0.26 (95% CI 0.14–0.50, *p* <0.0001) without increasing clinical failure and mortality ^61^. Readmission rates and subsequent exacerbations were similar in the two groups. As discussed previously, a reduction of antibiotic use is a critical component of AMS.

The most common bacterial organisms isolated in AECOPD remain *Haemophilus influenzae* and *S. pneumoniae*; however, *Moraxella catarrhalis* and the atypical organisms may also be seen ^62^. Viral infections may predispose patients to bacterial infections, and the specific bacteria isolated depend on factors such as age of more than 65 years, steroid use, comorbid illness such as cardiac disease, structural lung disease, or more severe COPD—Global Initiative for Chronic Obstructive Lung Disease (GOLD) 3–4 lung functions—and previous antibiotic use in the past 3 months (as would be the case in frequent exacerbators) ^63– 65^. In fact, some studies indicate that more resistant organisms such as *Klebsiella pneumoniae*, *Staphylococcus aureus*, *Pseudomonas aeruginosa*, *Escherichia coli*, and *Acinetobacter* and *Enterobacter* species are increasingly being seen, particularly in developing countries, even without these risk factors ^66– 69^. This implies that the choice of agent depends on both risk factors and local epidemiology. However, we would recommend that therapy be initiated with *H. influenzae* and *S. pneumoniae* in mind ^70^.

As such, the initial antibiotic choice would be amoxicillin (or amoxicillin–clavulanate where beta-lactamase production by *H. influenzae* is prevalent) or a fluoroquinolone. However, the US Food and Drug Administration has recommended that the latter be used only as a last resort agent and be reserved for use in patients who have no other treatment options because of both side effects and potential collateral damage ^71^.

Other agents, such as the cephalosporins cefuroxime or cefpodoxime, may be appropriate as they are active against *H. influenzae* and higher doses would also be effective against the pneumococcus. The extended-spectrum macrolides may be effective against the former but increasing resistance of the pneumococcus has limited their utility. For hospitalized patients with risks for pseudomonas or other more-resistant organisms, anti-pseudomonal agents such as piperacillin–tazobactam, cefepime, or ciprofloxacin may be considered. It is recommended that these patients have a sputum culture performed on admission ^72^.

The duration of the antibiotic “course” has been somewhat controversial in that, for a long time, there has been a mistaken belief that longer courses decrease resistance and improve outcome. This is patently untrue, and in many diseases (including COPD) shorter courses have had equivalent outcomes with fewer adverse events ^73^. As such, we would recommend 5 days as being the optimal duration for most AECOPD ^74– 76^.

The dosing of each antibiotic should be according to pharmacokinetic principles. The beta-lactams are time-dependent agents; as such, the target should be to exceed the minimum inhibitory concentration by as much time as possible, both to limit resistance and to improve outcome. The fluoroquinolones, as concentration-dependent agents, should preferably be administered once daily to achieve a maximal area under the inhibitory curve (AUIC) or an area under the curve-to-MIC ratio (AUC/MIC). Unfortunately, owing to toxicity issues, this is not possible with all agents in practice. For example, if an agent such as ciprofloxacin is going to be used, it must be administered twice daily because of suspected or documented infection with some of the Gram-negative pathogens described above ^77, 78^. The concept of “short-course high-dose antibiotic therapy” embodies these principles and should be employed wherever possible.

Of interest, established guidelines for the management of COPD exacerbations such as those of the GOLD initiative are generally not well adhered to and this includes antibiotic strategies. In a recent study evaluating compliance, 64 (68.1%) of the 94 patients received antibiotics, of which only 71.9% were appropriate ^79^.

Inhaled antibiotics have not been well evaluated; however, one study that used nebulized tobramycin twice daily for 14 days in patients with severe COPD colonized with multidrug-resistant *P. aeruginosa* demonstrated a 42% decrease in AECOPD compared with the previous 6 months and also a marked reduction in markers of inflammation, indicating a potential role for this modality of therapy ^80^.

An alternative use for antibiotics in patients with COPD has been prophylaxis for exacerbations, which are predictive of a worse outcome in COPD, and any means that might reduce them may potentially also improve long-term survival and quality of life ^81^. Numerous agents used for COPD have been shown to reduce exacerbations, and these include the long-acting antimuscarinic agents, inhaled CSs, and the phosphodiesterase 4 inhibitors. Antibiotics, specifically the macrolides, have also been shown to provide benefit; however, the exact mechanism has not been fully elucidated ^82– 84^. Different anti-inflammatory effects have been investigated *in vitro* but not proven *in vivo* ^85, 86^.

Doses vary, but the most frequent doses of azithromycin administered have been 250 or 500 mg three times a week. There are possible side effects from the use of these agents for this purpose, and these include resistance of bacteria and non-tuberculous mycobacteria, drug–drug interactions, QT prolongation, reversible deafness, and gastrointestinal upset ^85^. However, when used in selected patients, the macrolides are safe and cost effective ^86, 87^.

In line with stewardship principles, vaccination strategies, if effective, would be valuable in reducing antibiotic use. Whereas vaccination with the pneumococcal polysaccharide vaccine has provided inconsistent benefit in COPD, influenza vaccine—in particular, when combined with the pneumococcal polysaccharide vaccine (studies using the conjugate vaccine are not available but it would be expected to be better)—reduces hospitalization for pneumonia, death, death from influenza, and death from pneumonia ^88^. In addition, a meta-analysis of the use of an oral, whole-cell, non-typeable *H. influenzae* (NTHi) vaccine versus placebo in patients with AECOPD found a statistically significant 80% increase in antibiotic courses per person in the placebo group (risk ratio 1.81, 95% CI 1.35–2.44, *p* <0.0001) was noted ^89^.

## Conclusions

CAP and bacterial AECOPD are significant LRTIs associated with considerable morbidity and mortality. Despite numerous clinical studies as well as systematic reviews and meta-analyses, there is still ongoing debate as to the appropriate treatment of CAP, particularly in severely ill cases, and additionally which AECOPD actually need antibiotics. These issues are discussed in detail in the article, and recommendations are given on the basis of the authors’ collective experience. Furthermore, it is important to remember that, with regard to antibiotic use, these agents are a potentially life-saving resource that must be used wisely both in terms of the specific agent, duration, and dose and in the correct circumstances.
